# Outcomes of Epi-On Collagen Cross-Linkage Procedure Assessed in Progressive Keratoconus Patients

**DOI:** 10.7759/cureus.30664

**Published:** 2022-10-25

**Authors:** Sharmeen Akram, Sehrish Momin, Bilal Malik, Zubaida Sirang

**Affiliations:** 1 Department of Ophthalmology and Visual Sciences, Aga Khan University Hospital, Karachi, PAK

**Keywords:** vision, therapeutic interventions, corneal thickness, keratometry, collagen crosslinkage, corneal ectasia, keratoconus

## Abstract

Introduction: Keratoconus is a corneal ectasia that causes astigmatism and reduced vision. Conventional treatment to stop the progression of ectasia involves debridement of corneal epithelium, followed by ultraviolet light and riboflavin drops to reinforce the collagen covalent bonds, called collagen cross-linkage (CXL). Epi-on (epithelium-on) is a modified technique without epithelial debridement and associated complications of pain, infection, and damage to the cornea. However, despite a good safety index and efficacy, Epi-on has not completely replaced the conventional Epi-off (epithelium-off) CXL. We aim to report our five-year experience and outcomes with Epi-on CXL

Methods: In this five-year retrospective clinical audit, we included all patients who underwent Epi-on CXL from December 2014 to June 2020 at the Aga Khan University Hospital. Outcomes were based on best-corrected visual acuity (BCVA) and topographic indicators such as keratometry-max (K-max), keratometry mean (K-mean), pachymetry apex (Pach_apex_), and pachymetry thinnest (Pach_thin_) performed during pre-CXL clinical visit within one month of the procedure and were compared with the most remote follow up within three years post-CXL. A p-value of <0.05 was considered statistically significant.

Results: A total of 223 eyes of 134 patients had undergone CXL of which 32 eyes of 18 patients were included in the study based on the inclusion criteria. The mean age was 26.8 (+/- 6.137) years; nine were males and 16 were right eyes. Mean BCVA was 0.383 logMAR (logarithm of the minimum angle of resolution) units which improved to 0.292 units post CXL (p=0.02) and K-max decreased from 57.4 to 56.60 diopters (p=0.048), both outcomes were statistically significant. Pach_apex_ decreased slightly from 471 to 460 micrometers (p=0.099), K-mean was almost stable from 48.8 to 48.7 diopters (p=0.9), and Pach_thin_ also decreased slightly from 455 to 445 micrometers (p=0.117), however, these outcomes were not statistically significant. Other studies reported similar improvements in K-max and visual acuity.

Conclusion: Epi-on CXL is an effective treatment for halting the progression of keratoconus. Our results showed significant improvement in visual acuity and K-max readings indicating a halting of the progression of keratoconus in our patients. Long-term follow-up is required for all patients to assess detailed outcomes. Further studies comparing Epi-on CXL with other methods may be carried out.

## Introduction

Keratoconus is a corneal ectasia characterized by thinning of the corneal stroma. This causes astigmatism which results in a progressive, often asymmetrical decrease in vision. Its prevalence ranges from 0.6% to 2.3% in different populations [1]. Collagen cross-linking (CXL) of the progressively ectatic cornea is currently the mainstay of treatment to cease the progression of keratoconus. The treatment uses ultraviolet light and vitamin B2 (riboflavin) drops to re-enforce the corneal collagen fibrils by strengthening their covalent bonds [2]. The Dresden protocol described the conventional Epi-off (epithelium-off) CXL procedure in 2003, which involves mechanical debridement of the corneal epithelium [3]. However, the removal of epithelium increases the risk of endothelial damage, corneal infection, corneal hypoesthesia, sterile infiltrates, sub-epithelial haze, herpetic re-activation, corneal scarring, and delayed recovery of corneal sensitivity [4,5]. To overcome such complications, an improved method of epithelium on (Epi-on) CXL was introduced which does not involve epithelial debridement [2]. Although it has an acceptable safety index and has been very effective in halting disease progression, it has not yet replaced the Epi-off CXL procedure completely [6,7]. Very few studies have been published regarding the efficacy of the Epi-on procedure. This audit aims to describe the results in keratoconus patients following the Epi-on CXL procedure at a university hospital in Karachi, Pakistan.

## Materials and methods

We performed a retrospective clinical audit including all patients with progressive keratoconus who had undergone Epi-on CXL from December 2014 to June 2020 at the Aga Khan University Hospital, Karachi, Pakistan. Epi-on CXL was performed using a standard technique of instilling riboflavin drops (Collagex TransEpithelial), one drop every five minutes for 30 minutes, followed by exposure to irradiance of 3mW/cm^2^ ultraviolet-A light of 365nm (LightLink, LightMed Corp., CA, USA) for 30 minutes. Our target sample size was 30 eyes. Patients less than 18 years of age, those who had undergone Epi-off CXL or previous eye surgeries, and those with incomplete records or who were lost to follow-up were excluded from the study. Our primary outcome was a change in keratometry max (K-max) and secondary outcomes included best-corrected visual acuity (BCVA), change in keratometry mean (K-mean), pachymetry apex (Pach_apex_), and pachymetry thinnest (Pach_thin_).

We used a proforma to collect retrospective data with all relevant outcomes. The earliest clinic visit before CXL was taken as the pre-CXL visit, and the most recent clinic visit with a documented corneal topography was taken as the post-CXL visit. SPSS version 23 (IBM Corp., Armonk, NY) was used to analyze the data. Snellen’s visual acuity was converted to logMAR (logarithm of the minimum angle of resolution) values. Means with standard deviation and medians with interquartile ranges were computed to describe continuous data. Counts and proportions were computed to describe categorical data. A paired t-test was used to assess change in the continuous outcome variables. A p-value of <0.05 was considered statistically significant.

A formal exemption was obtained from the Ethical Review Committee of Aga Khan University (2020-5074-10996); all proformas and patient data were reviewed as per the confidential record-keeping policies of the institute. 

## Results

**Figure 1:**
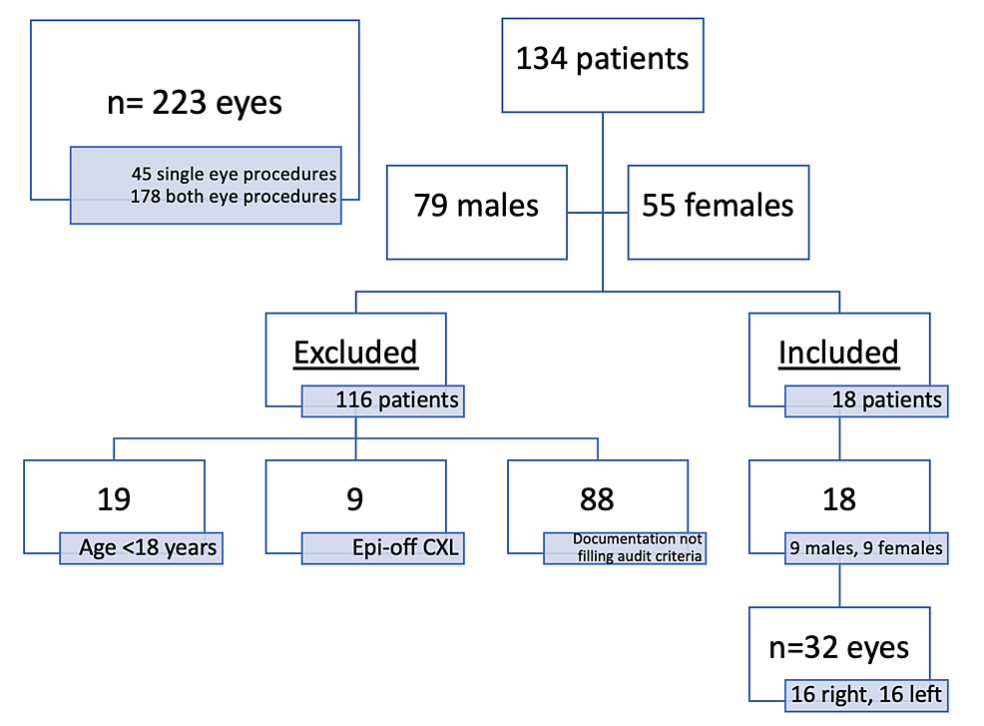
Flow chart for collagen cross-linkage (CXL) patients from 2014 till 2020

A total of 134 CXL patients were involved in CXL from December 2014 to June 2020. Of these, 45 patients had undergone a procedure on one eye only, hence a total of 223 eyes were assessed for eligibility criteria. A total of 32 eyes of 18 patients were included in our study based on the inclusion criteria and availability of the data from a retrospective chart review. The time range of the post-CXL assessment varied from a minimum of three months to up to three years (Figure 1).

**Figure 2:**
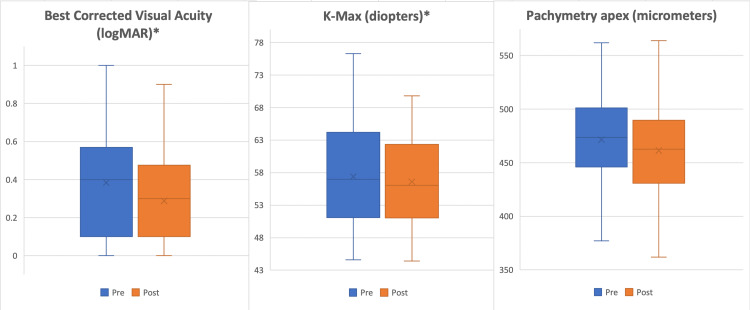
Box and whisker plot showing the comparison of pre-CXL and post-CXL values for BCVA, K-max, and Pachapex *statistically significant variables. (logMAR p-value 0.029, K-max p-value 0.048) CXL: collagen cross-linkage; BCVA: best-corrected visual acuity; K-max: keratometry-max; Pachapex: pachymetry apex.

Mean age of our patients was 26.78 years old. There were nine male patients and a total of 16 right eyes (Table 1). Outcome variables were assessed for normality using the Shapiro-Wilk test. The statistical differences between pre- and post-CXL variables are shown in Table 2. Paired t-test showed significantly lower K-max post CXL with a mean value of 56.6 (P= 0.048). The Pach_thin_ pre- and post-CXL remained stable with non-significant differences in mean pre-CXL (455.68) and post-CXL (445.24) thickness (P= 0.117). Pach_apex_ also remained reasonably stable with a pre-CXL mean of 471.50 and a post-CXL mean of 460.87 with no significant difference (P= 0.099). Wilcoxon signed-rank test was used to assess K-mean values and BCVA pre- and post-CXL as these were not normally distributed in our results. Signed-rank test elicited lower K-mean post-CXL (n= 15) and stable K-mean (n=4) showing improved outcomes in 19 patients (Z= -1.25, asymptotic significance P=0.90). A Wilcoxon signed-rank test elicited statistically significant improvement in visual acuity post treatment with 17 negative ranks representing 17 patients with improved logMAR visual acuity (Z= -2.18, asymptotic significance P=0.029). Box and whisker plot showing statistically significant improvements in BCVA and K-max is shown in Figure 2, along with stable Pach_apex_.

**Table 1:** Demographics and mean values of variables BCVA: best-corrected visual acuity; K-max: keratometry-max; K-mean: keratometry mean; Pachapex: pachymetry apex; Pachthin: pachymetry thinnest.

Gender	Male: 9	Female: 9
Eye	Right: 16	Left: 16
	Mean (+/- SD)	Range
Age (in years)	26.781 (+/- 6.137)	18 - 41
Pre-CXL characteristics		
BCVA (logMAR)	0.383 (+/- 0.277)	0.0 – 1.00
K-mean (diopters)	48.859 (+/- 4.0453)	42.10 – 59.20
K-max (diopters)	57.372 (+/- 7.926)	44.60 – 76.30
Pach_apex _(micrometers)	471.50 (+/- 38.424)	377 – 562
Pach_thin _(micrometers)	455.69 (+/- 42.130)	363 – 559
Post-CXL characteristics		
BCVA	0.292 (+/- 0.212)	0.00 – 0.90
K-mean	48.706 (+/- 4.323)	41.30 – 58.40
K-max	56.60 (+/- 7.230)	44.40 – 69.80
Pach_apex_	460.88 (+/- 46.343)	362 - 564
Pach_thin_	445.34 (+/- 44.624)	361 – 558

**Table 2:** Pre- and post-collagen cross-linkage (CXL) statistical differences a: non-normally distributed data, b: normally distributed data BCVA: best-corrected visual acuity; K-max: keratometry-max; K-mean: keratometry mean; Pachapex: pachymetry apex; Pachthin: pachymetry thinnest.

Outcomes (post CXL – pre CXL)							
Wilcoxon Signed Ranks^a^	Negative rank	Positive ranks	Ties	Sum of ranks			Z-score (A-symptotic P-value)
				Negative ranks		Positive ranks	
BCVA	17	7	4	225		74.5	-2.184 (0.029)
K-mean	15	13	4	208.5		108.5	-0.125 (0.900)
Paired T-test^b^	Mean (+/- SD)			95% confidence interval			P-value
				Lower		Upper	
K-max	0.77187 (+/- 2.117)			0.009	1.535		0.048
Pach_apex_	10.625 (+/- 35.356)			-2.122	23.372		0.099
Pach_thin_	10.344 (+/- 36.309)			-2.747	23.435		0.117

## Discussion

CXL to date remains the treatment of choice in progressive keratoconus, to bring a halt to the advancement of the disease process [8]. There are several methods of CXL for example epi-off, epi-on, and epi-on with iontophoresis [9]. Different indices have been used to assess the progression of keratoconus post-CXL treatment. Progression of keratoconus ectasia is defined as an increase of 1D or greater in the steepest K reading, an increase of 1D or greater in the manifest cylinder, or an increase of 0.5 D or more in spherical equivalent indicating progression of keratoconus. The most common indices reported to rate progression of keratoconus post-CXL are K-max, uncorrected distance visual acuity (UDVA), corrected distance visual acuity (CDVA), pachymetry apex, and pachymetry thinnest [10].

We report our clinical audit using the Epi-on CXL method using transepithelial riboflavin and UV light in 32 eyes. The prevalence of keratoconus, as mentioned is 0.6%-2.3%, and use of CXL is indicated for progressive keratoconus only [1]. Moreover, we excluded the paediatric population which further limited our sample size. Similar limitations are seen in various studies [6]. To assess the progression of the disease we used K-max, BCVA, Pach_apex_, Pach_thin_, and K-mean readings. A study by Stulting et al. showed a decrease in mean K-max by 0.48 D at two years postoperatively [11]. Another multi-center clinical trial conducted in the United States, showed K-max flattened by 1.6 diopters (D) in eyes with keratoconus and 0.7 D in eyes with post-refractive ectasia after one year [10]. Also, in a study by TG Seiler, similar results were found showing flattening of K-max one year post-CXL [12]. Another study of our local population demonstrated a significant reduction in K-max, steep K, and simulated K in the epi-on CXL group, as well as the epi-off group [7]. Our study results were similar to the above studies. Post-CXL our results showed lower K-max with a mean value of 56.6 D (P= 0.048), which represents a decrease of 0.8 D, from the pre-CXL mean of 57.4 D.

Stulting et al. showed in their study that the mean UDVA and CDVA improved by 1 to 1.5 Snellen lines at one and two years postoperatively (P<0.0001) [11]. Greenstein et al. also showed that BCVA improved by an average of 5.7 logMAR letters in the keratoconus treatment group and by 5.0 logMAR letters in the ectasia group [10]. The study in our local population also demonstrated improved mean UDVA and CDVA [7]. However, in a Cochrane Database review by Ng et al., eleven studies compared epi-on CXL with epi-off CXL in participants with progressive keratoconus. The results of the review showed that in epi-on CXL, little to no difference in CDVA at 12 months or beyond was noted [13]. In our audit, we observed no deterioration of visual acuity. In fact, there was a statistically significant improvement in visual acuity post-treatment (Z= -2.1, asymptotic significance P= 0.029). 

Also, in our study, the Pach_thin_ of cornea pre- and post-CXL remained stable with no significant differences in mean pre-CXL (455.68 microns) and mean post-CXL (445.24 microns) thickness (P= 0.117). Pach_apex_ remained stable as well with a pre-CXL mean of 471.50 microns and a post-CXL mean of 460.87 microns and with no significant difference between the two (P= 0.099). Similar pachymetry results were found in a study by Seiler [12]. Data from other studies in the local population showed corneal pachymetry at all test points with significantly greater reductions achieved in the epi-off CXL group at 18 months of follow-up [7].

The mainstay of CXL treatment is to halt the advancement of keratoconus in order to avoid a corneal transplant. Our audit result showed comparative results to other centers. However, limitations of our study included retrospective data collection, a single-centered study from a private hospital, multiple follow-up periods post-CXL based on the availability of the data, lack of review on patient co-morbids, and post-CXL complications. Larger sample size or multi-centered studies with prospective data collection and preferably randomised controlled trials are recommended for an accurate representation of the patient population.

## Conclusions

CXL is a hallmark treatment for halting the progression of keratoconus ectasia. Though the Epi-off technique is highly effective, the post-procedure risks are high. In comparison, Epi-on is an effective procedure showing promising results in improving visual and topographical parameters in keratoconus patients. Our results showed significant improvement in visual acuity and K-max readings, indicating halting of the progression of keratoconus in our patients. Long-term follow-up is required for all patients to assess detailed outcomes. Further prospective studies comparing Epi-on CXL with other methods are recommended. 
